# Prevalence and Predictors of Nonadherence to Treatment in Adult Patients Undergoing In‐Centre Haemodialysis in Public Dialysis Units: A Quantitative Cross‐Sectional Study

**DOI:** 10.1155/nrp/6510878

**Published:** 2026-09-24

**Authors:** Jawad Jameel Saleh, Maryam Alaradi

**Affiliations:** ^1^ Salmaniya Medical Complex, Manama, Bahrain, moh.gov.bh; ^2^ School of Nursing and Midwifery, Royal College of Surgeons in Ireland, Medical University of Bahrain, Busaiteen, Bahrain, uob.edu.bh

**Keywords:** adult, diet, fluid, haemodialysis, treatment adherence

## Abstract

**Background:**

Nonadherence to treatment regimens is common among patients on maintenance haemodialysis and has been well studied internationally and regionally; however, a local gap remains.

**Aim:**

This study aimed to determine the prevalence and predictors of treatment nonadherence among adults receiving in‐centre haemodialysis in Bahrain’s public units.

**Design:**

A cross‐sectional design.

**Participants:**

A total of 266 patients receiving haemodialysis were recruited from two public haemodialysis units using simple random sampling conducted independently within each study centre.

**Measurements Instrument:**

End Stage Renal Disease Adherence Questionnaire, demographics and clinical examination and laboratory results sheet.

**Results:**

The nonadherence rate to haemodialysis prescription was 85%, with 81.9% shortening sessions and 15.1% missing sessions in the previous month. Medication, fluid and dietary nonadherence rates were 18%, 29.3% and 20.6%, respectively. Bivariate analysis showed that participants aged > 60 years had better adherence to fluid restriction (*p* = 0.031) and medications (*p* = 0.006), while those treated at Centre A demonstrated higher adherence to haemodialysis prescriptions (*p* = 0.001) and medication therapy (*p* = 0.034). Regression model identified age as an independent predictor of medication adherence; participants aged > 60 years had 2.74 higher odds of adherence compared with those aged ≤ 60 years (*p* = 0.004). Participants attending Centre B had 53% lower odds of medication adherence than those at Centre A (*p* = 0.045). Accompanying participants, transportation issues and receipt of health education were not significantly associated with adherence across treatment domains.

**Conclusion:**

Nonadherence to haemodialysis treatment regimens varied across treatment domains among the study population. Healthcare providers in Bahrain’s public haemodialysis units should consider adherence behaviours and associated factors during patient assessment and follow‐up. Further studies are required to better understand treatment nonadherence among haemodialysis patients in public dialysis units and in Bahrain.

**Summary**
• What is already known about this topic?
◦ Adherence to the prescribed haemodialysis treatment regimen remains a significant challenge among patients receiving maintenance haemodialysis.
◦ The prevalence and factors associated with treatment adherence among haemodialysis patients have not previously been investigated in Bahrain.
◦ Poor adherence to treatment may adversely affect patients’ physical, mental and psychological health and their health‐related quality of life.
• What does this paper add?
◦ This is the first study in Bahrain to report on the prevalence of nonadherence to the treatment regimen among patients undergoing in‐centre haemodialysis.
◦ The findings support the need for routine assessment of adherence behaviours and patient‐specific barriers within haemodialysis care.
◦ The study may inform the development of targeted interventions to improve treatment adherence among haemodialysis patients in Bahrain.
◦ Results establish baseline evidence to support future research on treatment adherence in Bahrain’s haemodialysis population.
◦ The findings may support nursing education by enhancing the understanding of some of the treatment adherence challenges among the haemodialysis population.
• Implications of this study:
◦ Results of this study may enhance understanding of nonadherence phenomena in patients receiving haemodialysis in Bahrain.
◦ Inform the development of a structured policy for assessing treatment adherence among patients receiving in‐centre haemodialysis, ultimately improving patient outcomes and guiding evidence‐based nursing practice.
◦ Findings may guide tailoring patient‐targeted intervention based on age and the dialysis centre in Bahrain.

## 1. Introduction

Chronic kidney disease (CKD) is a major global health burden, affecting an estimated 674 million individuals across stages 1–5, with the majority residing in low‐ and middle‐income countries [1]. Also, it was estimated that approximately 3.9 million individuals are receiving renal replacement therapy [2]. This number is subject to an increase to approximately 5.439 million by 2030 [3]. Al Agha, Al Qaseer [4] reported that in Bahrain, 536 patients with ESRD received renal replacement therapy in the public dialysis unit in 2020. Haemodialysis was the most dominant treatment modality in Bahrain in 2020. Among the patients with ESRD who received renal replacement therapy in the public dialysis unit of Bahrain in 2020, 478 (89.2%) of patients were treated with regular in‐centre haemodialysis, while 58 (10.8%) of patients underwent chronic peritoneal dialysis [4]. More recent service utilisation data from the Abdulrahman Kanoo Centre for Kidney Diseases indicate that 44,776 in‐centre haemodialysis sessions were conducted in 2022 [5].

End‐stage renal disease (ESRD) occurs when the estimated glomerular filtration rate drops below 15 mL/kg/min. At this stage, renal function is insufficient for human survival and requires renal replacement therapy. Diabetes, hypertension and cardiovascular disease are among the common risk factors associated with CKD and ESRD [6]. The Global Burden of Disease study identified high fasting plasma glucose (31.9%), high systolic blood pressure (24.5%) and high body mass index (23.5%) as the leading risk factors contributing to the global burden of CKD [7]. In Bahrain, the high prevalence of hypertension (33.6%) and diabetes mellitus (14.7%) may contribute to the burden of CKD [8].

McKay and Verhagen [9] conceptualised adherence as an ongoing and dynamic process that is profoundly influenced by the surrounding environment. This perspective recognises that individuals’ health‐related behaviours are not only a function of their personal knowledge, motivation, skills and available resources but are also shaped by broader social contexts. Adherence explains why patients do not pursue a specific recommendation by their healthcare providers as advised and why patients might use recommendations more or less than recommended [9, 10].

Nonadherence to the treatment regimen is common among patients receiving haemodialysis [11–14]. Nonadherence to treatment regimen may be linked to gender [13], educational level [15, 16], financial status [17] and type of vascular access [13]. The consequences of nonadherence may be far‐reaching and multifaceted, encompassing physical, mental and psychological impact and changes in health‐related quality of life [18–20].

While the existing literature has extensively explored the nonadherence problem among patients on maintenance haemodialysis [14, 18, 21, 22], locally, there remains a notable gap in understanding the prevalence and factors of this problem. Thus, this study is essential for establishing new knowledge about the nonadherence phenomenon in Bahrain. Also, the study allows for a deeper insight into the factors influencing adherence to treatment regimen among haemodialysis patients and possibly enables the nursing team/multidisciplinary team in the in‐centre dialysis unit to develop strategies to educate and facilitate ease of treatment, thus enhancing the overall development of care for patients.

### 1.1. Aim

This study aimed to assess the prevalence of nonadherence to the treatment regimen and identify predictors of nonadherence to the treatment regimen among adult patients undergoing in‐centre haemodialysis in Bahrain’s public haemodialysis units.

### 1.2. Objectives

1. To assess the prevalence of nonadherence among patients undergoing haemodialysis in public dialysis units in Bahrain.
2. To identify predictors of nonadherence among patients undergoing haemodialysis in public dialysis units in Bahrain.

### 1.3. Research Questions

1. What is the nonadherence prevalence among adult haemodialysis patients in Bahrain’s Public haemodialysis units?
2. What are the predictors of nonadherence to treatment regimens in adult patients undergoing haemodialysis in Bahrain’s public haemodialysis units?

### 1.4. Theoretical Framework

The Health Belief Model (HBM) was used as a theoretical framework to evaluate patients’ adherence to the treatment regimen. The HBM includes the constructs of perceived susceptibility, perceived severity, perceived benefits, perceived barriers and cues to action [23]. Demographics within the HBM form patients’ perceptions [24]. HBM constructs were believed to predict nonadherence behaviours among haemodialysis patients.

This study utilised all HBM constructs except self‐efficacy, as evaluating patients’ confidence in self‐care management was beyond the scope of the study. The constructs of perceived barriers and cues to action were assessed through questionnaire items examining factors related to transportation, dialysis schedule convenience, accompaniment during haemodialysis sessions and receipt of health education, which were analysed in relation to treatment adherence among individuals undergoing maintenance haemodialysis in Bahrain’s public dialysis units. Perceived susceptibility, perceived severity and perceived benefits were also assessed through the study instrument; however, these construct‐level findings are not presented in the main manuscript. The corresponding questionnaire items and results are provided in the supporting information (Supporting File 1).

## 2. Methods

### 2.1. Design

A descriptive cross‐sectional study design was employed and reported in accordance with the Strengthening the Reporting of Observational Studies in Epidemiology (STROBE) guidelines.

### 2.2. Study Setting

This study was conducted in two public haemodialysis units serving the majority of patients undergoing haemodialysis in Bahrain. The first dialysis unit is located in a large public hospital in Bahrain in Manama (Centre A). It serves adult and paediatric patients. Also, it is the primary dialysis unit, where the newly diagnosed patients receive their haemodialysis session at an early stage. The unit operates 24 h a day. The second dialysis centre is located in northern Bahrain (Centre B). It serves adult patients only. It operates 16 h per day. Centres A and B function under the same organisation’s umbrella.

### 2.3. Study Population and Sampling

The study population consisted of adult patients receiving treatment at two public in‐centre haemodialysis units, for a total of 500 patients. Centre A had 190 patients, while Centre B had 310. All patients were on a haemodialysis schedule of either two sessions per week or more. Patients aged ≥ 18 years at both centres were eligible to participate, except those with documented cognitive impairment that prevented them from providing informed consent or understanding the questionnaire items. Eligibility was verified by reviewing patients’ medical records before the sampling process began. Additionally, non‐Arabic‐speaking patients were excluded because the adopted study instrument was in Arabic, and comprehension issues could compromise data reliability. After applying the inclusion and exclusion criteria, 405 patients were eligible for participation, 135 from Centre A and 270 from Centre B. Accordingly, independent sample size calculations were conducted for each centre, using the Raosoft online calculator, applying a 95% confidence level and a 5% margin of error. An additional 10% was added to each sample to account for potential nonresponses or incomplete questionnaires. This approach resulted in sample sizes reflecting centre‐level representation rather than proportional allocation relative to the total eligible population. This was necessary to ensure sufficient representation of Centre A due to its smaller patient population and distinct operational and patient characteristics, including a higher burden of comorbidities and lower overall health status compared to Centre B, more scheduled haemodialysis shifts and the inclusion of patients receiving haemodialysis during night sessions. These factors may influence patient experiences and adherence behaviours, thus warranting sampling within each centre to allow robust analysis. The sample calculation yielded 113 participants from Centre A and 174 from Centre B for a total of 287 participants across both centres. Then, a sequentially numbered sampling frame of eligible patients was constructed at each centre, and subject selection was conducted using simple random sampling through an online number generator, with number duplication not permitted.

### 2.4. Data Collection

Demographic data, clinical examination and laboratory results sheet, and End Stage Renal Disease Adherence Questionnaire (ESRD AQ) were used to collect data for this study. The authors used these data to assess the prevalence of nonadherence and identify its related factors.

#### 2.4.1. Demographic Data

Demographic data collected include gender, age, dialysis centre and dialysis schedule.

#### 2.4.2. Clinical Examination and Laboratory Results Sheet

Inter‐dialytic weight gain (IDWG), attendance at the dialysis unit, duration of haemodialysis sessions and pre‐dialysis serum potassium and phosphorus levels were collected by pre‐assigned nurses acting as gatekeepers. These clinical indicators were utilised to assess the prevalence of nonadherence to the prescribed haemodialysis treatment regimen.

Subjects were considered nonadherent to fluid restriction when IDWG was greater than 5.7% at least once during the week before the survey [25]. Patients were considered nonadherent to haemodialysis prescription if they missed at least one session or shortened any session by ≥ 10 min during the preceding month [18, 19]. Changes in prescriptions during hospitalisation or shortening sessions due to documented interdialytic complications were excluded. Nonadherence to dietary recommendations was evaluated using the most recent pre‐dialysis serum potassium levels. A level exceeding 6.0 mmol/L indicated poor adherence to dietary restrictions [25, 26]. Medication adherence was assessed via serum phosphorus levels, with values greater than 2.4 mmol/L considered indicative of nonadherence to phosphate binder usage [25, 27].

#### 2.4.3. ESRD AQ

The ESRD AQ was developed by Kim et al. [28] to produce a valid and reliable instrument that assesses adherence behaviours of patients receiving in‐centre haemodialysis in four treatment dimensions. It includes questions related to haemodialysis prescription, medication use and following fluid and dietary recommendations. The ESRD AQ consists of 46 questions divided into five sections. The first section of the treatment dimension includes questions related to the history of diseases and renal replacement therapy. The four sections include questions about adherence to haemodialysis prescription, medication, fluid restriction and dietary recommendations. The instrument assesses the patient’s knowledge and perception of treatment. Questions in the instrument utilise a combination of Likert scales, multiple choice and a ‘yes and no’ answer format. The instrument was tested for validity and reliability and found to be valid with a content validity index (CVI) of 0.86–1 in all instrument items and reliable in test and re‐test stability with an intra‐class correlation of 0.83–1 in all instrument items. The ESRD AQ is publicly available for use without cost via the PubMed database [28].

The original English version of the questionnaire was translated into Arabic and then back‐translated to ensure lexical equivalence. The translated instrument was tested for validity [25]. The Arabic version was requested and obtained from the author, and permission was obtained to use the instrument for this study.

#### 2.4.4. Data Collection Procedure

Participants were recruited over 8 weeks, from 1 April to 31 May 2024. Pre‐assigned gatekeepers obtained written informed consent from interested subjects. Consenting participants completed a self‐administered ESRD AQ. The survey completion time ranged from 30 to 40 min. If subjects were tired, they were allowed to pause and continue later. The intention was to distribute the survey during the first hour of haemodialysis so that the survey could be completed before the occurrence of interdialytic hypotension, exhaustion and fatigue towards the end of the session. The gatekeepers completed the clinical examination and laboratory result sheet from the electronic medical record.

#### 2.4.5. Data Analysis

The data were analysed using IBM SPSS version 26. A statistician specialist conducted the analysis. The sample characteristics and traits were described using frequency distribution, central tendency and variability. Inferential analysis was performed to identify the factors associated with nonadherence. First, the chi‐squared test was used to identify the association between study variables and adherence to the haemodialysis treatment regimen. Then, variables with *p*‐values < 0.05 or considered to be clinically or theoretically relevant were entered into a binary logistic regression model to identify independent predictors of nonadherence. This predictive approach enabled the calculation of adjusted odds ratios (AORs) to identify independent predictors of nonadherence within the HBM framework. Continuous variables in the study included age, IDWG, serum potassium level and serum phosphorus level. Age was analysed both as a continuous variable for descriptive statistics and as a categorical variable for inferential analysis, with categories of ≤ 60 and > 60 years. Categorical variables included gender, dialysis unit, haemodialysis schedule, timing of the last health education session and length of haemodialysis treatment. Adherence was analysed as a binary outcome (adherent vs. nonadherent) in all inferential analyses. Data completeness was verified before analysis. Missing data were minimal: two responses were missing for fluid adherence, one for adherence to dialysis attendance and one for adherence to prescribed haemodialysis duration. Missing values were imputed prior to analysis using an appropriate method determined by a statistical specialist. Sampling weights were not incorporated in the analysis, as the study employed centre‐specific sample allocation to enhance representation and ensure adequate participant inclusion from each haemodialysis centre.

## 3. Results

A total of 266 participants participated in the study. Only 21 (7.3%) of the invited subjects did not consent to participate in the study. Participants were recruited from both dialysis centres, with a greater proportion from Centre B (61.3%). The sample showed a slight predominance of males and participants aged over 60 years, with a mean age of 58.6 years (SD = 14.7). Both dialysis schedules were represented, with a slight predominance of participants following the Saturday, Monday and Wednesday schedules (Table 1).

**TABLE 1:** Frequencies and percentages of the subjects’ demography (*N* = 266).

Variable	Categories	Count	%
Gender	Male	157	59.0
	Female	109	41.0

Age	≤ 60 years	128	48.1
	> 60 years	138	51.9

Centre	SMC	103	38.7
	ARK	163	61.3

### 3.1. Disease and Dialysis Profile

Most participants reported a haemodialysis treatment duration of up to 5 years (71.1%). Haemodialysis was the primary renal replacement therapy for the majority (98.2%). While previous kidney transplantation was reported by relatively few participants (6.7). Personal transportation was the most common mode of travel to the dialysis centre (83.1%), and nearly half of the participants attended dialysis independently. Most participants received haemodialysis three times per week (92.5%) and generally reported their dialysis schedule as convenient (91.4%) (Table 2).

**TABLE 2:** Frequencies and percentages of the subjects’ disease and dialysis profile (*N* = 266).

Variable	Categories	*n* (%)
When did you begin your haemodialysis treatment?	< 1 year	34 (12.8)
	1–5 years	155 (58.3)
	6–10 years	56 (21.1)
	> 10 years	21 (7.9)

Did you restart haemodialysis before?	No	261 (98.2)
	Yes	5 (1.8)

Have you ever had chronic peritoneal dialysis treatment?	No	246 (92.5)
	Yes	20 (7.5)

Have you had a kidney transplant?	No	249 (93.6)
	Yes	17 (6.4)

What type of transportation do you use to go to the dialysis centre?	Personal transport	221 (83.1)
	Bus	1 (0.4)
	Taxi	5 (1.9)
	Hospital escort	36 (13.5)
	Other	3 (1.1)

Who accompanies you to the dialysis centre?	Myself	127 (47.7)
	Parents	7 (2.6)
	Wife/husband	29 (10.9)
	Son/daughter	59 (22.2)
	Friend	6 (2.3)
	Other	2 (0.8)
	Brother/sister	12 (4.5)
	Maid/driver	22 (8.3)
	Cousin/in‐law	2 (0.8)

How many days a week do you receive haemodialysis treatment?	Two times or less	13 (4.9)
	Three times	246 (92.5)
	Four times	6 (2.3)
	> 4 times	1 (0.4)

How many hours are you treated for each haemodialysis?	< 3 h	3 (1.1)
	3 h	97 (36.4)
	3 h 15 min	2 (0.8)
	3 h 30 min	83 (31.2)
	3 h 45 min	5 (1.9)
	4 h	76 (28.6)

Is your dialysis schedule convenient for you?	Yes	243 (91.4)
	No, because I have to come early	3 (1.1)
	No, because I have to come late	17 (6.4)
	No, because of my work schedule	1 (0.4)
	Other	2 (0.8)

### 3.2. Prevalence of Nonadherence to the Treatment Regimen

The mean dry body weight of the study participants was 73.4 ± 17.2 kg. The mean IDWG across the last three haemodialysis sessions was 3.5 ± 6.6, 3.8 ± 7.9 and 4.8 ± 12.5 kg, respectively. The mean pre‐dialysis serum potassium level was 5.4 ± 3.7 mmol/L, while the mean phosphorus level was 1.9 ± 0.7 mmol/L.

Nonadherence to the duration of the haemodialysis session was prevalent. A large proportion of participants shortened at least one dialysis session by 10 min or more within the past month without documented medical approval (81.9%). Although less frequent, session absenteeism was also noted, where a minority of participants had missed at least one scheduled dialysis session during the preceding month (15.1%). Overall, nonadherence to the prescribed dialysis regimen was high, with the majority of participants deviating from the recommended session frequency or duration (85%). Regarding medication therapy, a minority of participants were nonadherent to prescribed medications (18%). Nonadherence to dietary recommendations was also a minority finding (20.6%), while nonadherence to fluid restrictions represented a substantial proportion of participants (29.3%).

### 3.3. Factors Associated With Adherence to Haemodialysis Prescription

Examining the factors associated with nonadherence to the haemodialysis prescription using a chi‐squared test revealed that adherence to the haemodialysis prescription differed significantly by dialysis centre, with patients treated at Centre A demonstrating higher adherence compared with those at Centre B (*p* = 0.001). Also, older participants exhibited a greater tendency to adhere to the prescribed regimen than those younger than 60 years. However, this difference was not statistically significant (*p* = 0.231) (Table 3). Full descriptive and chi‐squared results are presented in Supporting File 1. To further examine these associations, a binary logistic regression analysis was conducted to identify predictors of adherence to the haemodialysis prescription. The overall regression model was not statistically significant (*p* = 0.061). Among the examined predictors, dialysis centre was the only variable that reached statistical significance in the regression model, with participants at Centre B having 68.7% lower odds of adherence to haemodialysis prescription compared with participants in Centre A (*p* = 0.002). None of the remaining predictors demonstrated statistically significant associations with adherence to haemodialysis prescription (Table 4). Full binary logistic regression results are presented in Supporting File 1.

**TABLE 3:** Chi‐squared analysis of factors associated with adherence to haemodialysis prescription (*N* = 266).

Study variables	Adherent *n (%)*	*p*
Subject gender		0.728
Male	25 (15.9)	
Female	15 (13.8)	
Dialysis centre		0.001^∗^
A	25 (24.3)	
B	15 (9.2)	
Age group		0.231
≤ 60 years	96 (75.0)	
60 years	122 (88.4)	
Schedule		0.308
SMW	24 (17.3)	
STT	16 (12.6)	
Convenience of dialysis schedule		0.727
Yes	37 (15.2)	
No, because I have to come late	3 (17.6)	
Who accompanied you to the dialysis centre?		0.399
Myself	23 (18.)	
Family member	13 (11.9)	
Other	4 (13.3)	
Type of transportation to dialysis centre		0.168
Personal	30 (13.6)	
Other	10 (22.2)	
Length of haemodialysis treatment		0.175
1 year	4 (11.8)	
1–5 years	21 (13.5)	
6–10 years	8 (14.3)	
10 years	7 (33.3)	
Last health education		0.803
Never	10 (14.1)	
This week	4 (12.5)	
Last weeks	2 (9.5)	
Last month	4 (11.4)	
> 1 month	7 (22.6)	
Since started the haemodialysis	11 (15.1)	

^∗^ *p* value using *chi*‐squared test ≤ 0.05 was statistically significant.

**TABLE 4:** Binary logistic regression analysis of factors associated with adherence to haemodialysis prescription (*N* = 266).

Variable	AOR	95% CI	*p*
Gender	0.729	0.399–1.59	0.425
Dialysis centre	0.313	0.15–0.65	0.002**^∗^**
Age group	0.624	0.29–1.32	0.221
Schedule	0.844	0.40–1.77	0.653
Convenience of dialysis schedule	0.839	0.48–1.46	0.536
Who accompanies you to the dialysis centre?	1.033	0.87–1.22	0.710
Type of transportation	1.139	0.82–1.56	0.426
Length of haemodialysis treatment	1.365	0.84–2.20	0.202
Last health education	1.065	0.88–1.28	0.512

*Note:* Omnibus test = 0.061, Cox andSnell *R* ^2^ = 0.060 and Nagelkerke *R* ^2^ = 0.107. Bold formatting indicates statistically significant values.

^∗^ *p* value using binary logistic regression ≤ 0.05 was statistically significant.

### 3.4. Factors Associated With Adherence to Medication Therapy

Chi‐squared analysis revealed statistically significant associations between adherence to medication therapy and both age group and dialysis centre. Participants aged ≤ 60 years were significantly less adherent compared to those > 60 years (*p* = 0.006). Similarly, adherence was higher among patients from Centre A when compared with those of Centre B (*p* = 0.034). Although patients on the Saturday–Monday–Wednesday (SMW) schedule reported higher adherence compared to those on the Sunday–Tuesday–Thursday (STT) schedule, the difference was not statistically significant (*p* = 0.057) (Table 5). Full descriptive and chi‐squared results are presented in Supporting File 1.

**TABLE 5:** Chi‐squared analysis of factors associated with adherence to medication therapy (*N* = 266).

Study variables	Adherent *n* (%)	*p*
Subject gender		0.630
Male	127 (80.9)	
Female	91 (83.5)	
Age group		0.006**^∗^**
≤ 60 years	96 (75.0)	
> 60 years	122 (88.4)	
Dialysis unit		0.034**^∗^**
A	91 (88.3)	
B	127 (77.9)	
Schedule		0.057
SMW	120 (86.3)	
STT	98 (77.2)	
Length of haemodialysis treatment		0.863
< 1 year	29 (85.3)	
1–5 years	128 (82.6)	
6–10 years	44 (78.6)	
> 10 years	17 (81.0)	
Last health education		0.453
Never	74 (83.1)	
This week	20 (87)	
Last weeks	11 (91.7)	
Last month	45 (75)	
> 1 month	31 (88.6)	
Since started the haemodialysis	37 (78.7)	

*Note:* Bold formatting indicates statistically significant values.

^∗^ *p* value using *chi*‐squared test ≤ 0.05 was statistically significant.

A binary logistic regression analysis was conducted to examine independent predictors associated with adherence to medication therapy. The regression model was statistically significant (*p* = 0.004). Age was an independent predictor of medication adherence. Patients aged > 60 years had 2.74 higher odds of adherence compared to those aged ≤ 60 (*p* = 0.004). The dialysis centre was also an independent predictor of medication adherence, with participants attending Centre B having 53% lower odds of adherence compared with those attending Centre A (*p* = 0.045). Other assessed predictors were not significantly associated with medication therapy adherence (Table 6). Full binary logistic regression results are presented in Supporting File 1.

**TABLE 6:** Binary logistic regression analysis of factors associated with adherence to medication therapy (*N* = 266).

Variable	AOR	95% CI	*p*
Schedule	0.551	0.285–1.065	0.076
Gender	1.056	0.540–2.064	0.874
Age	2.739	1.392–5.386	0.004**^∗^**
Dialysis centre	0.470	0.225–0.984	0.045**^∗^**
Last health education	1.006	0.846–1.196	0.947
Constant	3.090		0.348

*Note:* Omnibus test = 0.004, Cox and Snell *R* ^2^ = 0.062 and Nagelkerke *R* ^2^ = 0.102. Bold formatting indicates statistically significant values.

^∗^ *p* value using binary logistic regression ≤ 0.05 was statistically significant.

### 3.5. Factors Associated With Adherence to Dietary Recommendations

The chi‐squared test showed that none of the examined variables was significantly associated with adherence to dietary recommendations among patients on haemodialysis (Table 7). Full descriptive and chi‐squared results are presented in Supporting File 1. To further examine these associations, a binary logistic regression analysis was conducted to identify independent predictors of adherence to the dietary recommendations. The overall model failed to achieve statistical significance (*p* = 0.285). Furthermore, an evaluation of the individual variables within the final equation confirmed that none of the five examined variables was a statistically significant predictor of adherence to dietary recommendations (Table 8). Full binary logistic regression results are presented in Supporting File 1.

**TABLE 7:** Chi‐squared analysis of factors associated with adherence to dietary recommendation (*N* = 266).

Study variables	Adherent *n* (%)	*p*
Subject gender		0.123
Male	130 (48.9)	
Female	81 (30.5)	
Age group		0.453
≤ 60 years	99 (37.2)	
> 60 years	112 (42)	
Dialysis unit		0.163
A	77 (28.9)	
B	134 (50.4)	
Schedule		0.546
SMW	108 (77.7)	
STT	103 (81.1)	
Length of haemodialysis treatment		0.169
< 1 year	30 (88.2)	
1–5 years	125 (80.6)	
6–10 years	39 (69.6)	
> 10 years	17 (81)	
Last health Education		0.209
Never	54 (87.1)	
This week	29 (80.6)	
Last weeks	17 (77.3)	
Last month	25 (71.4)	
> 1 month	23 (71.9)	
Since started the haemodialysis	62 (79.5)	

*Note:* *p* value using chi‐squared test ≤ 0.05 was statistically significant.

**TABLE 8:** Binary logistic regression analysis of factors associated with adherence to dietary recommendations (*N* = 266).

Variable	AOR	95% CI	*p*
Schedule	0.144	0.625–2.133	0.646
Gender	−0.539	0.316–1.077	0.085
Age	0.274	0.710–2.433	0.384
Dialysis centre	0.366	0.772–2.694	0.251
Last health education	−0.065	0.780–1.125	0.486
Constant	0.889		0.414

*Note:* Omnibus test = 0.285, Cox and Snell *R* ^2^ = 0.023 and Nagelkerke *R* ^2^= 0.36. *p* value using binary logistic regression ≤ 0.05 was statistically significant.

### 3.6. Factors Associated With Adherence to Fluid Restriction Recommendations

Among the variables analysed using the chi‐squared test, only the age group was significantly associated with adherence to fluid restriction recommendations. Participants aged > 60 years demonstrated higher adherence compared to those aged ≤ 60 years (*p* = 0.031) (Table 9). Full descriptive and chi‐squared results are presented in Supporting File 1. A binary logistic regression analysis was conducted following bivariate screening to identify independent predictors of adherence to fluid restriction recommendations. The overall model did not reach statistical significance (*p* = 0.086). Age category was the only statistically significant predictor of fluid restriction adherence (*p* = 0.018), with participants aged > 60 years having approximately 94% higher odds of adherence compared to those aged ≤ 60 years. No other predictors achieved statistical significance within the equation (Table 10). Full binary logistic regression results are presented in Supporting File 1.

**TABLE 9:** Chi‐squared analysis of factors associated with adherence to fluid restriction recommendation (*N* = 266).

Study variables	Adherent *n* (%)	*p*
Subject gender		0.891
Male	110 (41.4)	
Female	78 (29.3)	
Age group		0.031**^∗^**
≤ 60 years	82 (30.8)	
> 60 years	106 (30.8)	
Dialysis unit		0.168
A	78 (29.3)	
B	110 (41.4)	
Schedule		0.139
SMW	104 (74.8)	
STT	84 (66.1)	
Length of haemodialysis treatment		0.783
< 1 year	22 (64.7)	
1–5 years	109 (70.3)	
6–10 years	41 73.2)	
> 10 years	16 (76.2)	
Last health education		0.600
Never	37 (88.1)	
This week	5 (62.5)	
Last weeks	10 (76.9)	
Last month	39 (78)	
> 1 month	52 (77.6)	
Since started the haemodialysis	68 (80)	

*Note:* Bold formatting indicates statistically significant values.

^∗^ *p* value using *chi*‐squared test ≤ 0.05 was statistically significant.

**TABLE 10:** Binary logistic regression analysis of factors associated with adherence to fluid restriction recommendations (*N* = 266).

Variable	AOR	95% CI	*p*
Schedule	0.663	0.384–1.144	0.140
Gender	0.996	0.569–1.744	0.989
Age	1.935	1.122–3.338	0.018**^∗^**
Dialysis centre	0.674	0.380–1.197	0.178
Last health education	1.011	0.880–1.162	0.877
Constant	1.602		0.622

*Note:* Omnibus test = 0.086, Cox and Snell *R* ^2^  = 0.036 and Nagelkerke *R* ^2^  = 0.051. Bold formatting indicates statistically significant values.

^∗^ *p* value using binary logistic regression ≤ 0.05 was statistically significant.

## 4. Discussion

This study aimed to assess the prevalence of nonadherence to the treatment regimen and identify its influencing factors among adults on maintenance haemodialysis.

### 4.1. Study Sample Characteristics

The final sample size was 266 subjects, 38.7% from Centre A and 61.3% from Centre B. Among the study sample, 59% were males and 41% were females. The sample mean age was 58.6 ± 14.7 years. The required sample size for each centre was achieved, enhancing the representativeness of participants recruited from both haemodialysis units. The mean age of 58.6 ± 14.7 is lower than the sample from Turkey (62.57 ± 13.24) and the United States (63.6 ± 14.6) [13, 14]. The younger mean age may reflect Bahrain’s high prevalence of diabetes (14.7%) and hypertension (33.6%) [8]. In addition, younger individuals may underestimate ESRD risks, while sociocultural expectations on men to work and provide for their family may delay health‐seeking, increasing advanced CKD and ESRD cases.

### 4.2. Prevalence of Nonadherence to Treatment

The study results show a variation in the nonadherence rate between treatment domains. While it was as high as 85% for the haemodialysis prescription, it was lower for adherence to fluid restriction recommendation (29.3%), dietary recommendations (20.6%) and medication therapy (18%). A high prevalence of nonadherence reflects poor follow‐up of self‐care recommendations. In contrast, a low prevalence of nonadherence reflects better follow‐up of the self‐care recommendation provided by the care team in the in‐centre dialysis units.

On the one hand, this study reported a lower level of nonadherence to fluid restriction recommendations (29.3%) compared with the rates reported in Indonesia (40.5%) and Nepal (51.1%) [29, 30]. Similarly, the reported nonadherence rate to medication therapy (18%) is lower than the reported rate in Denmark (32%) and Turkey (20.1%) [12, 13]. Locally, the nonadherence rate to medication therapy among study subjects (18%) is lower than that among patients with other chronic diseases who follow in the primary care setting (80.5%) [31]. Moreover, the current study found that nonadherence to dietary recommendations among patients in the public haemodialysis unit was 20.6%, which is substantially lower than the international prevalence rate of 60.2% [14]. On the other hand, the results of the current study indicate that nonadherence to haemodialysis prescription was 85%, which is markedly higher than the rates reported in Makkah City, Saudi Arabia (44%) and Turkey (33.6%) [13, 25].

The comparison of adherence rates between the current study and previous studies has limitations due to the differences in study settings and measurement tools or the adopted cutoff point, where a lower cutoff point may lead to an inflated rate for nonadherence.

### 4.3. Factors Associated With Nonadherence

Results from the chi‐squared test demonstrated that participants aged over 60 demonstrated significantly higher adherence to fluid restriction and medication therapy compared to those aged 60 years or younger. Additionally, patients receiving haemodialysis at Centre A were more likely to adhere to both haemodialysis prescriptions and medication therapy than those at Centre B. Binary logistic regression confirms that age and dialysis centre were independent predictors of medication adherence after controlling for confounders. Age and dialysis centre were also associated with fluid restriction and haemodialysis prescription adherence in the regression level; however, the overall models did not reach statistical significance, suggesting limited explanatory power and the potential influence of unmeasured confounding variables for both haemodialysis prescription and fluid restriction recommendation domains. Social support and transportation issues were not significantly associated with haemodialysis prescription adherence, and receipt of health education was not associated with adherence across treatment domains in either chi‐squared or regression analyses.

On the one hand, the study results are consistent with other studies’ results. Al‐Khattabi, Baljoon [25] reported that older haemodialysis patients in Makkah City, Saudi Arabia, demonstrated a higher level of adherence to fluid restriction compared with younger patients. Also, Bazrafshan, Darvizeh [32] reported that older age was found to be independently associated with better medication adherence among haemodialysis patients in Iran. Older age was an independent risk factor for nonadherence to fluid restriction [33]. On the other hand, in Turkey, age was not found to be associated with adherence to either medication or fluid restriction recommendations [13]. Similarly, age did not influence adherence to medication therapy in Makkah city, Saudi Arabia [25]. Locally, Al‐Sayyad, Alshehabi [31] reported that age was not associated with adherence to medication therapy among patients with chronic diseases.

In contrast to the current study result, the literature frequently reported a positive association between social support and adherence to haemodialysis sessions [25, 34]. Similarly, transportation has been widely reported as a major barrier to adherence to haemodialysis sessions [18, 35–37], whereas in this study, transportation issues were not significantly associated with adherence to haemodialysis prescription. Moreover, literature emphasises the role of education in improving adherence to the treatment regimen [36, 38, 39], while the current study’s recent educational session did not achieve statistical significance.

A possible explanation for older subjects’ higher adherence to medication and fluid restrictions is that younger individuals may perceive themselves at lower risk for disease complications. Their busy lifestyles and work or family obligations can further hinder adherence. In contrast, older adults often maintain more structured routines that align better with treatment requirements and help prevent complications.

The difference in adherence to medication therapy between Centre A and Centre B was supported by both bivariate and regression analyses, with dialysis centre confirmed as a predictor of medication adherence after controlling for confounders. This may be attributed to the internal policy of patient referral between the two centres, whereby patients with a lower disease burden are referred to Centre B, while those with a higher disease burden remain at Centre A. Patients with a higher disease burden may perceive greater susceptibility to complications and severity of symptoms, potentially motivating higher adherence [24]. Regarding haemodialysis prescription adherence, the association with dialysis centre was identified at the bivariate level, with Centre A demonstrating higher adherence compared with Centre B; however, the overall regression model did not achieve statistical significance, limiting the ability to confirm dialysis centre as a predictor in this domain. Therefore, the interpretation of centre‐related differences in haemodialysis prescription adherence should be approached cautiously and may warrant further investigation incorporating additional variables.

The observed inconsistency in social support may be a result of defining it solely as accompanying subjects during haemodialysis sessions. However, social support also includes emotional encouragement, help with daily activities, transportation, dietary management and decision‐making assistance.

### 4.4. Strengths and Limitations

On the one hand, the study demonstrates several strengths. First, the findings address a significant gap in the literature regarding the adherence of haemodialysis patients in Bahrain. Second, using an objective approach to assess the prevalence of nonadherence to treatment increased the validity of the results and minimised the risk of recall or social desirability bias. On the other hand, several limitations should be considered when interpreting the findings. First, the use of a centre‐specific sample size calculation approach may limit the generalisability of the findings. Although this approach was methodologically justified to ensure adequate representation of Centre A, the final sample does not reflect the natural distribution of patients across centres. Sampling weights were not applied, as the study was not intended to produce population‐level estimates; therefore, findings should be interpreted as reflecting associations within the study sample rather than as population‐representative estimates. Second, the sample size was determined based on estimating prevalence and was not powered for analytical modelling. Accordingly, the results of the chi‐squared tests and multivariable regression analysis should be interpreted with caution. Third, the cross‐sectional design limits the ability to detect causal relationships or capture changes in adherence over time. Fourth, a small number of participants were bed‐bound (*n* = 2, 0.75%), and intradialytic weight gain was estimated by nurses following clinical assessment due to the absence of bed scales in the units. This may introduce measurement inaccuracy. Finally, the exclusion of non‐Arabic‐speaking participants may limit the external validity and generalisability of the findings in the haemodialysis population of Bahrain, which is linguistically diverse.

## 5. Study Implication

The findings of this study may encourage nurses in in‐centre dialysis units to obtain a comprehensive patient history to assess treatment adherence, identify its factors and implement early corrective interventions. In addition, the results could inform the development of new policies within the public dialysis units to assess and monitor nonadherence behaviours.

## 6. Conclusion

This cross‐sectional study examined adherence across treatment domains among patients undergoing in‐centre haemodialysis in Bahrain. The study results revealed varied levels of adherence across the treatment domains. Older participants demonstrated significantly higher adherence to fluid restriction and medication therapy than younger patients. Moreover, patients treated at Centre A were more likely to adhere to their haemodialysis prescriptions and medications. HBM provided a robust framework for interpreting the results. The study’s results fill a critical knowledge gap in the research on adherence in patients undergoing maintenance haemodialysis in Bahrain and have direct implications for haemodialysis care. Finally, further research is required to establish consistency about the nonadherence phenomenon in haemodialysis patients in Bahrain.

## Author Contributions

Both authors made substantial contributions to the study. The specific contributions, based on the CRediT taxonomy, are as follows: (1) Jawad Jameel Saleh: conceptualisation, methodology development, data collection and writing original draft. (2) Maryam Alaradi: supervision, review and validation and writing review and editing.

## Funding

This study was conducted as part of the requirements for the Master of Nursing Science program at the Royal College of Surgeons in Ireland—Medical University of Bahrain (RCSI Bahrain). The authors received no specific grant or financial support for the purpose of this research. All costs related to data collection, analysis and manuscript preparation were covered by the authors.

## Disclosure

Patients, service users or members of the public were not involved in the design, conduct, reporting or dissemination plans of this research. However, the study data collected from haemodialysis patients and the findings will be shared with the participating dialysis units.

Statistical Compliance Statement Statistical analysis was performed by Mr Redha Abdulla Al‐Hammam, Expert Statistician, Government Hospitals, Bahrain. He was responsible for data screening, handling missing data and verifying the accuracy of the final analysis. Mr Al‐Hammam can be contacted at RHamman@hospitals.gov.bh.

## Ethics Statement

Ethical approvals for the study were obtained from the Royal College of Surgeons in Ireland—Bahrain Research Ethics Committee, approval number REC/2023/210/05‐Dec‐2023 and the Government Hospitals Research and Ethics Committee, approval number 126261223.

The study was conducted in accordance with the Declaration of Helsinki. Written informed consent was obtained from all participants. Participation was entirely voluntary. Participants were informed of their right to withdraw from the study without facing any negative consequences or disruption to their care. The authors could not link surveys to the participants. All data were handled in accordance with the Bahrain Personal Data Protection Law No. 30 of 2018 and the European Union’s General Data Protection Regulation, ensuring strict compliance with data security and privacy standards.

## Conflicts of Interest

The authors declare no conflicts of interest.

## Supporting Information

Additional supporting information can be found online in the Supporting Information section.

## Supporting information


**Supporting Information 1** Descriptive, chi‐squared and binary logistic regression results for the study sample.

**Supporting Information 2** STROBE Statement—checklist of items that should be included in reports of observational studies.

## Data Availability

The data that support the findings of this study are available from the corresponding author upon reasonable request.
