# Supplementary material for: Prevalence and Predictors of Nonadherence to Treatment in Adult Patients Undergoing In‐Centre Haemodialysis in Public Dialysis Units: A Quantitative Cross‐Sectional Study
Source: Nurs Res Pract. 2026 Sep 24;2026:6510878. doi: 10.1155/nrp/6510878 (PMC13602352; doi:10.1155/nrp/6510878)
Supplement: Supplementary file 2 [file NRP-2026-6510878-s002.docx]

**Supplementary File 1:**

***Frequencies and percentages of treatment non-adherence (N = 266)***

| **Treatment Domain** | **Count** | **%** |
| --- | --- | --- |
| Non-adherence to the haemodialysis duration | 218 | 81.9 |
| Non-adherence to the haemodialysis schedule | 40 | 15.1 |
| Overall non-adherence to haemodialysis prescription (duration and schedule) | 226 | 85 |
| Non-adherence to medication therapy | 48 | 18 |
| Non-adherence to dietary recommendations | 55 | 20.6 |
| Non-adherence to fluid restriction | 78 | 29.3 |

**Frequencies and Percentages of the Subjects' History of Haemodialysis Schedule (N = 266)**

| **Question** | **Response** | **n (%)** |
| --- | --- | --- |
| When was the last time a medical professional (your doctor, nurse, dietician, or other medical staff) talked to you about the importance of not missing your dialysis treatment? | This week | 32 (12.0) |
|  | Last weeks | 21 (7.9) |
|  | Last month | 35 (13.2) |
|  | More than a month ago | 31 (11.7) |
|  | Since I started the haemodialysis | 73 (27.4) |
|  | Never | 71 (26.7) |
|  | Other | 3 (1.1) |
| How often does a medical professional (your doctor, nurse, dietician, or other medical staff) talk to you about the importance of staying for the entire dialysis time during your dialysis treatment? | Each haemodialysis session | 24 (9.0) |
|  | Every week | 16 (6.0) |
|  | Every month | 39 (14.7) |
|  | 2 to 3 months | 22 (8.3) |
|  | 4 to 6 months | 5 (1.9) |
|  | When labs are not normal | 12 (4.5) |
|  | Rarely | 56 (21.1) |
|  | Not regularly | 20 (7.5) |
|  | Never | 71 (26.7) |
|  | Other | 1 (0.4) |
| How important do you think it is to follow your dialysis schedule? | Highly important | 223 (83.8) |
|  | Very important | 26 (9.8) |
|  | Moderately Important | 8 (3.0) |
|  | A little important | 8 (3.0) |
|  | Not important | 1(0.4) |
| Why do you think it is important to follow your dialysis schedule? | Because I fully understand that my kidney condition requires dialysis as scheduled | 76 (28.6) |
|  | Because following the dialysis schedule is important to keep my body healthy | 147 (55.3) |
|  | Because a medical professional told me to do so | 10 (3.8) |
|  | Because I had an experience that I was sick after I missed dialysis | 25 (9.4) |
|  | Because I had an experience that I was hospitalised after I missed dialysis | 3 (1.1) |
|  | I don't think following the dialysis schedule is very important to me | 5 (1.9) |
| How much difficulty have you had staying for your entire dialysis treatment as ordered by your doctor? | No difficulty | 97 (36.5) |
|  | Slight difficulty | 38 (14.3) |
|  | Moderate difficulty | 41 (15.4) |
|  | Big difficulty | 23 (8.6) |
|  | Extreme difficulty | 67 (25.2) |
| **Question** | **Response** | **n (%)** |
| During the last month, how many dialysis treatments did you miss completely? | None | 219 (82.3) |
|  | 1 session | 22 (8.3) |
|  | 2 sessions | 11(4.1) |
|  | 3 sessions | 6 (2.3) |
|  | ≥4 sessions | 8 (3.0) |
| What was the main reason you missed your dialysis treatment last month? | N/A | 219 (82.3) |
|  | Transport issues | 4 (1.5) |
|  | Busy with other things | 11 (4.1) |
|  | Committed to an appointment | 3 (1.1) |
|  | Visiting A/E | 2 (0.8) |
|  | I was admitted | 5 (1.9) |
|  | Forgot | 1 (0.4) |
|  | Didn't have the desire to go for haemodialysis | 19 (7.1) |
|  | Other | 2 (0.8) |
| Why didn't you want to go to the dialysis centre? | I had vomiting‎/diarrhoea | 2 (10) |
|  | Muscular cramp | 1 (5.0) |
|  | Exhausted | 12 (60) |
|  | Had another sickness | 2 (10) |
|  | Depressed | 1 (5.0) |
|  | Other | 2 (10.0) |

**Frequencies and Percentages of the Subjects' History of Haemodialysis Session Duration (N = 266)**

| **Question** | **Response** | **n (%)** |
| --- | --- | --- |
| During the last month, how many times have you shortened your dialysis time? | N/A | 63 (23.7) |
|  | 1 time | 8 (3.0) |
|  | 2 times | 10 (3.8) |
|  | 4 times | 8 (3.0) |
|  | 5 to 6 times | 26 (9.8) |
|  | Other | 151(56.8) |
| During the last month, when your dialysis treatment was shortened, what was the average number of minutes? | Never | 60 (22.6) |
|  | ≤10 mins | 8 (3.0) |
|  | 11-20 mins | 9 (3.4) |
|  | 21-30 mins | 91 (34.2) |
|  | >30 mins | 94 (35.3) |
|  | Other | 4 (1.5) |
| What was the main reason you have shortened your dialysis treatment? | Never | 57 (21.4) |
|  | Muscular cramp | 11 (4.1) |
|  | Toileting | 1 (0.4) |
|  | Anxiety | 3 (1.1) |
|  | Hypotension | 20 (7.5) |
|  | Appointment with doctor | 3 (1.1) |
|  | Personal commitments | 20 (7.5) |
|  | Work commitments | 3 (1.1) |
|  | Transport issues | 14 (5.3) |
|  | Order from HPCs | 5 (1.9) |
|  | Do not want to stay | 113 (42.5) |
|  | Other | 16 (6.0) |

**Frequencies and Percentages of the Subjects' History of Medication Therapy (N = 266)**

| **Questions** | **Response** | **n (%)** |
| --- | --- | --- |
| When was the last time a medical professional (your doctor, nurse, dietician or other medical staff) spoke to you about your medicines? | This week | 23 (8.6) |
|  | Last weeks | 12 (4.5) |
|  | Last month | 60 (22.6) |
|  | More than a month | 35 (13.2) |
|  | Since I started the haemodialysis | 47 (17.7) |
|  | Never | 89 (33.5) |
| How often does a medical professional (your doctor, nurse, dietician or other medical staff) talk to you about the importance of taking medicines as ordered? | Each haemodialysis session | 7 (2.6) |
|  | Every week | 10 (3.8) |
|  | Every month | 57 (21.4) |
|  | 2 to 3 months | 22 (8.3) |
|  | 4 to 6 months | 8 (3.0) |
|  | When labs are not normal | 16 (6.0) |
|  | Rarely | 31 (11.7) |
|  | Not regularly | 12 (4.5) |
|  | Never | 102 (38.3) |
|  | Other | 1 (0.4) |
| How important do you think it is to take your medicines as scheduled? | Highly important | 200 (75.2) |
|  | Very important | 36 (13.5) |
|  | Moderately Important | 17 (6.4) |
|  | A little important | 5 (1.9) |
|  | Not important | 8 (3.0) |
| Why do you think it is important to take your medicines as scheduled? | Because I fully understand that my kidney condition requires to take medicines as scheduled | 72 (27.1) |
|  | Because taking medicines is important to keep my body healthy | 153 (57.5) |
|  | Because a medical professional told me to do so | 10 (3.8) |
|  | Because I had an experience that I was sick after I missed medicines | 8 (3.0) |
|  | I don't think taking medicine is very important to me | 22 (8.3) |
|  | Other | 1 (0.4) |
| Have you had any difficulty with taking your medicines? | No | 171 (64.3) |
|  | Yes | 95 (35.7) |
| How much difficulty have you had with taking your prescribed medicines? | No difficulty | 180 (67.7) |
|  | Little difficulty | 40 (15.0) |
|  | Moderate difficulty | 22 (8.3) |
|  | A lot of difficulty | 12 (4.5) |
|  | Extreme difficulty | 12 (4.5) |
| During the past week, how often have you missed your prescribed medicines? | None of the time | 150 (56.4) |
|  | Very seldom | 60 (22.6) |
|  | About half of the time | 26 (9.8) |
|  | Most of the time | 9 (3.4) |
|  | All the time | 21 (7.9) |
| What was the main reason for not taking your prescribed medicines this past week? | Not applicable: I did not miss medicines | 158 (59.4) |
|  | Forgot to take medicines | 36 (13.5) |
|  | Forgot to order medicines | 3 (1.1) |
|  | Inconvenience | 33 (12.4) |
|  | Side effects | 25 (9.4) |
|  | Other | 11 (4.1) |
| What kind of side effect(s) to the medication(s) did you have? | Nausea/vomiting/diarrhoea/constipation | 14 (56.0) |
|  | Stomach pain | 5 (20.0) |
|  | Dizziness | 2 (8.0) |
|  | Itching‎/skin problem | 1 (4.0) |
|  | Other | 3 (12.0) |

**Frequencies and Percentages of the Subjects' History of Fluid Restriction (N = 266)**

| **Question** | **Response** | **n (%)** |
| --- | --- | --- |
| When was the last time a medical professional (your doctor, nurse, dietician or other medical staff) spoke to you about your fluid restrictions? | This week | 36 (13.5) |
|  | Last week | 22 (8.3) |
|  | One month ago | 35 (13.2) |
|  | More than a month ago | 32 (12.0) |
|  | When I began haemodialysis | 78 (29.3) |
|  | Never | 62 (23.3) |
|  | Other | 1 (0.4) |
| How often does a medical professional (your doctor, nurse, dietician or other medical staff) talk to you about the importance of fluid restriction? | Every dialysis treatment | 24 (9.0) |
|  | Every week | 32 (12.0) |
|  | Every month | 37 (13.9) |
|  | Every 2-3 months | 13 (4.9) |
|  | Every 4-6 months | 10 (3.8) |
|  | When I have abnormal blood or other (for example, blood pressure) test | 39 (14.7) |
|  | Rarely | 32 (12.0) |
|  | Irregularly | 22 (8.3) |
|  | Never | 57 (21.4) |
| During the past week, how often have you followed the fluid restriction recommendations? | All the time | 124 (46.6) |
|  | Most of the time | 60 (22.6) |
|  | About half of the time | 30 (11.3) |
|  | Seldom | 12 (4.5) |
|  | None of the time | 40 (15.0) |
| How important do you think it is to limit your fluid intake? | Highly important | 212 (79.7) |
|  | Very important | 30 (11.3) |
|  | Moderately Important | 12 (4.5) |
|  | A little important | 4 (1.5) |
|  | Not important | 8 (3.0) |
| Why do you think it is important for you to limit your fluid intake? | Because I fully understand that my kidney condition requires limiting fluid intake | 55 (22.9) |
|  | Because limiting fluid intake is important to keep my body healthy | 133 (50.2) |
|  | Because a medical professional told me to do so | 6 (2.3) |
|  | Because I got sick after I drank lots of fluid | 54 (20.4) |
|  | Because I was hospitalised after I drank lots of fluid | 6 (2.3) |
|  | I don't think limiting fluid is very important to me | 11 (4.2) |
| **Question** | **Response** | **n (%)** |
| Have you had any difficulty with limiting your fluid intake? | No | 135 (50.8) |
|  | Yes | 131 (49.2) |
| How much difficulty have you had following your fluid restriction recommendations? | No difficulty | 127 (47.7) |
|  | Little difficulty | 44 (16.5) |
|  | Moderate difficulty | 46 (17.3) |
|  | A lot of difficulty | 28 (10.5) |
|  | I was unable to follow any recommendations at all | 21 (7.9) |
| If you had difficulty following your fluid restriction recommendations, what type of difficulty have you had? | No difficulty | 134 (50.6) |
|  | Not interested | 8 (3.0) |
|  | I was unable to control fluid intake | 114 (43.0) |
|  | I don't understand how to follow the fluid restriction | 7 (2.6) |
|  | Other | 2 (0.8) |
| During the past week, how many times have you weighed yourself at home (outside dialysis centre) | More than 3 times | 46 (17.3) |
|  | 3 times | 14 (5.3) |
|  | Twice | 17 (6.4) |
|  | Once | 15 (5.6) |
|  | None | 174 (65.4) |
| How important do you think it is to weigh yourself daily? | Highly important | 95 (35.7) |
|  | Very important | 22 (8.3) |
|  | Moderately Important | 18 (6.8) |
|  | A little important | 8 (3.0) |
|  | Not important | 123 (46.2) |

**Frequencies and Percentages of the Subjects' History of Dietary Recommendations (N = 266)**

| **Question** | **Response** | **n (%)** |
| --- | --- | --- |
| When was last time a medical professional (your doctor, nurse, dietician, or other medical staff) talked to you about your diet? | This week | 8 (3.0) |
|  | Last week | 13 (4.9) |
|  | One month ago | 50 (18.9) |
|  | More than a month ago | 67 (25.3) |
|  | When I began haemodialysis | 85 (32.1) |
|  | Never | 42 (15.8) |
| How often does a medical professional (your doctor, nurse, dietician or other medical staff) talk to you about the importance of following a proper diet? | Every dialysis treatment | 5 (1.9) |
|  | Every week | 7 (2.6) |
|  | Every month | 36 (13.5) |
|  | Every 2-3 months | 21 (7.9) |
|  | Every 4-6 months | 18 (6.8) |
|  | When I have abnormal blood or other test | 22 (8.3) |
|  | Rarely | 65 (24.4) |
|  | Irregularly | 16 (6.0) |
|  | Never | 73 (27.4) |
|  | Other | 3 (1.1) |
| How important do you think it is to watch the types of food you eat each day? | Highly important | 154 (58.1) |
|  | Very important | 53 (20.0) |
|  | Moderately Important | 21 (7.9) |
|  | A little important | 8 (3.0) |
|  | Not important | 29 (10.9) |
| Why do you think it is important for you to watch your diet daily? | Because I fully understand that my kidney condition requires me to watch my diet | 46 (17.1) |
|  | Because watching my diet is important to keep my body healthy | 157 (59.2) |
|  | Because a medical professional told me to do so | 8 (3.0) |
|  | Because I got sick after eating certain foods that I was not supposed to eat | 8 (3.0) |
|  | Because I was hospitalized after eating certain foods that I was not supposed to eat | 2 (0.8) |
|  | I don't think watching my diet is important to me | 44 (16.6) |
| Have you had any difficulty following your dietary recommendations? | No | 142 (53.4) |
|  | Yes | 124 (46.6) |
| How much difficulty have you had following your dietary recommendations? | No difficulty | 124 (46.6) |
|  | Little difficulty | 48 (18.0) |
|  | Moderate difficulty | 42 (15.8) |
|  | A lot of difficulty | 22 (8.3) |
|  | I was unable to follow any recommendations at all | 30 (11.3) |
| **Question** | **Response** | **n (%)** |
| What type of difficulty have you had keeping your dietary recommendations? | No difficulty | 132 (49.6) |
|  | I was not willing to control what I wanted to eat | 24 (9.0) |
|  | I was unable to avoid certain unrecommended food | 87 (32.7) |
|  | I don't understand what type of diet to follow | 23 (8.6) |
| During the past week, how many times have you followed the diet recommendations? | All the time | 79 (29.7) |
|  | Most of the time | 92 (34.6) |
|  | About half of the time | 36 (13.5) |
|  | Seldom | 17 (6.4) |
|  | None of the time | 42 (15.8) |

**Association Between Non-adherence to Haemodialysis Prescription, Medication Therapy, Dietary Recommendations, Fluid Restriction, and Dialysis Schedule with Adherence to Treatment Regimen. Using Chi-Square Test (N = 266)**

| **Variables** | **Haemodialysis prescription** | | **Medication Therapy** | | **Dietary recommendation‎** | | **Fluid restriction** | |
| --- | --- | --- | --- | --- | --- | --- | --- | --- |
|  | **Adherent n (%)** | ***p*** | **Adherent n (%)** | ***P*** | **Adherent n (%)** | ***P*** | **Adherent n (%)** | ***P*** |
| **Gender: Male Female** | 25 (15.9) | .728 | 127 (80.9) | .630 | 130 (48.9) | .123 | 110 (41.4) | .891 |
|  | 15 (13.8) |  | 91(83.5) |  | 81(30.5) |  | 78(29.3) |  |
| **Age group ≤ 60 years > 60 years** | 23 (18.0) | .231 | 96 (75.0) | **.006*** | 99 (37.2) | .453 | 82 (30.8) | **.031*** |
|  | 17 (12.3) |  | 122 (88.4) |  | 112 (42) |  | 106 (30.8) |  |
| **Dialysis Unit A B** | 25 (24.3) | **.001*** | 91(88.3) | **.034*** | 77 (28.9) | .163 | 78 (29.3) | .168 |
|  | 15 (9.2) |  | 127 (77.9) |  | 134 (50.4) |  | 110 (41.4) |  |
| **Schedule SMW STT** | 24 (17.3) | .308 | 120 (86.3) | .057 | 108 (77.7) | .546 | 104 (74.8) | .139 |
|  | 16 (12.6) |  | 98 (77.2) |  | 103 (81.1) |  | 84 (66.1) |  |

* P-value using *Chi*-Square test ≤ .05 was Statistically significant.

**Association Between Study Variables and Adherence to Haemodialysis Prescription. Using Chi-Square Test (N = 266)**

| **Study Variables** | **Adherent *n (%)*** | ***P*** |
| --- | --- | --- |
| **Convenience of dialysis schedule Yes No, because I have to come late** | 37 (15.2) | .727 |
|  | 3 (17.6) |  |
| **Who accompanies you to the dialysis centre? Myself Family member Other** | 23 (18.) | .399 |
|  | 13 (11.9) |  |
|  | 4 (13.3) |  |
| **Type of transportation Personal Other** | 30 (13.6) | .168 |
|  | 10 (22.2) |  |

**Association Between Study Variables and Adherence to Haemodialysis Treatment Regimen. Using Chi-Square Test (N = 266)**

| **Study variables** | **Haemodialysis prescription** | | **Medication use** | | **Dietary recommendation** | | **Fluid restriction** | |
| --- | --- | --- | --- | --- | --- | --- | --- | --- |
|  | **Adherent n (%)** | **p** | **Adherent n (%)** | **P** | **Adherent n (%)** | **P** | **Adherent n (%)** | **P** |
| **Length of hemodialysis treatment** <1 year 1-5 years 6-10 years >10 years | 4 (11.8) | .175 | 29 (85.3) | .863 | 30 (88.2) | .169 | 22 (64.7) | .783 |
|  | 21 (13.5) |  | 128 (82.6) |  | 125 (80.6) |  | 109 (70.3) |  |
|  | 8 (14.3) |  | 44 (78.6) |  | 39 (69.6) |  | 41 73.2) |  |
|  | 7 (33.3) |  | 17 (81.0) |  | 17 (81) |  | 16 (76.2) |  |
| **Last health Education** Never This week Last weeks Last month >1month Since started the haemodialysis | 10 (14.1) | .803 | 74 (83.1) | .453 | 54 (87.1) | .209 | 37(88.1) | .600 |
|  | 4 (12.5) |  | 20 (87) |  | 29 (80.6) |  | 5 (62.5) |  |
|  | 2 (9.5) |  | 11 (91.7) |  | 17 (77.3) |  | 10 (76.9) |  |
|  | 4 (11.4) |  | 45 (75) |  | 25 (71.4) |  | 39 (78) |  |
|  | 7 (22.6) |  | 31 (88.6) |  | 23 (71.9) |  | 52 (77.6) |  |
|  | 11 (15.1) |  | 37 (78.7) |  | 62 (79.5) |  | 68 (80) |  |

* P-value using *Chi*-Square test ≤ .05 was Statistically significant.

Table: 6

Chi-Square Analysis of Factors Associated with Adherence to Medication Therapy (N = 266)

| **Study Variables** | **Adherent *n (%)*** | ***P*** |
| --- | --- | --- |
| Subject gender  Male  Female | 127 (80.9)  91(83.5) | .630 |
| Length of hemodialysis treatment  <1 year  1-5 years  6-10 years  >10 years | 29 (85.3)  128 (82.6)  44 (78.6)  17 (81.0) | .863 |
| Last health Education  Never  This week  Last weeks  Last month  >1month  Since started the haemodialysis | 74 (83.1)  20 (87)  11 (91.7)  45 (75)  31 (88.6)  37 (78.7) | .453 |

* P-value using *Chi*-Square test ≤ .05 was Statistically significant

|  | **Binary logistic regression analysis of factors associated with haemodialysis prescription adherence (N 266)** | | | | | | | | |
| --- | --- | --- | --- | --- | --- | --- | --- | --- | --- |
| Variable | | B | S.E. | Wald | df | Sig. | AOR(Exp(B)) | 95% CI |  |
| Gender | | -.317 | .397 | .636 | 1 | .425 | .729 | 0.33-1.59 |  |
| Dialysis Centre | | -1.162 | .380 | 9.343 | 1 | .002* | .313 | 0.15-0.66 |  |
| Age | | -.471 | .385 | 1.500 | 1 | .221 | .624 | 0.29-1.32 |  |
| Schedule | | -.170 | .378 | .202 | 1 | .653 | .844 | 0.40-1.77 |  |
| Convenience of dialysis schedule | | -.176 | .284 | .382 | 1 | .536 | .839 | 0.48-1.46 |  |
| Who accompanies you to the dialysis centre? | | .033 | .088 | .139 | 1 | .710 | 1.033 | 0.87-1.22 |  |
| Type of transportation | | .130 | .163 | .635 | 1 | .426 | 1.139 | 0.82-1.56 |  |
| Length of haemodialysis treatment | | .311 | .244 | 1.627 | 1 | .202 | 1.365 | 0.84-2.20 |  |
| Last health education | | .063 | .096 | .430 | 1 | .512 | 1.065 | 0.88-1.28 |  |
| Constant | | .874 | 1.460 | .358 | 1 | .549 | 2.397 |  |  |

* P-value using binary logistic regression ≤ .05 was Statistically significant.

Omnibus test = .061, Cox & Snell R²=0.060, Nagelkerke R²=0.107.

***Binary logistic regression analysis of factors associated with adherence to medication therapy (N 266)***

|  | B | S.E. | Wald | df | Sig. | AOR(Exp(B)) | 95% C.I. | |
| --- | --- | --- | --- | --- | --- | --- | --- | --- |
|  |  |  |  |  |  |  | Lower | Upper |
| Schedule | -.597 | .336 | 3.145 | 1 | .076 | .551 | .285 | 1.065 |
| Gender | .054 | .342 | .025 | 1 | .874 | 1.056 | .540 | 2.064 |
| Age | 1.007 | .345 | 8.522 | 1 | .004* | 2.739 | 1.392 | 5.386 |
| Dialysis Centre | -.754 | .377 | 4.008 | 1 | .045* | .470 | .225 | .984 |
| Last health education | .006 | .088 | .004 | 1 | .947 | 1.006 | .846 | 1.196 |
| Constant | 1.128 | 1.201 | .882 | 1 | .348 | 3.090 |  |  |

* P-value using binary logistic regression ≤ .05 was Statistically significant.

Omnibus test = .004, Cox & Snell R²=.062, Nagelkerke R²=.102

| **Binary logistic regression analysis of factors associated with adherence to dietary recommendations (N 266)** | | | | | | | | | | | | | | | | | |
| --- | --- | --- | --- | --- | --- | --- | --- | --- | --- | --- | --- | --- | --- | --- | --- | --- | --- |
|  | B | | S.E. | | Wald | | df | | Sig. | | Exp(B) | | 95% C.I. | | | |  |
|  |  |  |  |  |  |  |  |  |  |  |  |  | Lower | | Upper | |  |
| schedule | | .144 | | .313 | | .211 | | 1 | | .646 | | 1.155 | | .625 | | 2.133 |  |
| Gender | | -.539 | | .313 | | 2.964 | | 1 | | .085 | | .583 | | .316 | | 1.077 |  |
| Age | | .274 | | .314 | | .759 | | 1 | | .384 | | 1.315 | | .710 | | 2.433 |  |
| Dialysis centre | | .366 | | .319 | | 1.319 | | 1 | | .251 | | 1.442 | | .772 | | 2.694 |  |
| Last health education | | -.065 | | .093 | | .486 | | 1 | | .486 | | .937 | | .780 | | 1.125 |  |
| Constant | | .889 | | 1.089 | | .668 | | 1 | | .414 | | 2.434 | |  | |  |  |

* P-value using binary logistic regression ≤ .05 was Statistically significant.

Omnibus test = .285, Cox & Snell R²=.023, Nagelkerke R²=.36

| **Binary logistic regression analysis of factors associated with adherence to fluid restriction recommendation (N 266)** | | | | | | | | | | | | | |  |
| --- | --- | --- | --- | --- | --- | --- | --- | --- | --- | --- | --- | --- | --- | --- |
|  | | B | S.E. | Wald | | df | | Sig. | AOR (Exp(B)) | | 95% C.I. | | | |
|  |  |  |  |  |  |  |  |  |  |  | Lower | Upper | | |
| Schedule | -.412 | | .279 | | 2.180 | | 1 | .140 | | .663 | .384 | | 1.144 | |
| Gender | -.004 | | .286 | | .000 | | 1 | .989 | | .996 | .569 | | 1.744 | |
| Age group | .660 | | .278 | | 5.627 | | 1 | .018 | | 1.935 | 1.122 | | 3.338 | |
| Dialysis Centre | -.394 | | .293 | | 1.812 | | 1 | .178 | | .674 | .380 | | 1.197 | |
| Last health education | .011 | | .071 | | .024 | | 1 | .877 | | 1.011 | .880 | | 1.162 | |
| Constant | .471 | | .956 | | .243 | | 1 | .622 | | 1.602 |  | |  | |
| * P-value using binary logistic regression ≤ .05 was Statistically significant.  Omnibus test = .086, Cox & Snell R²=.036, Nagelkerke R²=.051 | | | | | | | | | | | | | |  |
